# The Role of Social Media in Aesthetic Medicine Education ‐ A Global Analysis of Publication Habits and Scientific Impact

**DOI:** 10.1111/jocd.70984

**Published:** 2026-06-14

**Authors:** Giovanni Buzzaccarini, Victor R. M. Munoz‐Lora, Marcelo Germani, Pietra Roschel Borba, Mariana Shimizu, Anezka Juniese Albornoz Barraza, Martin Rodrigo Sepúlveda Candia, Sergio Escobar, Kristina Davidovic, Dmitry V. Melnikov, Carlos Bravo, Ishaan Ramkisson, Marco Borin, Giulio Nittari, Rebecca Susanna Degliuomini, Sebastian Cotofana

**Affiliations:** ^1^ Obstetrics and Gynaecology Unit, IRCCS San Raffaele Scientific Institute Vita‐Salute San Raffaele University Milan Italy; ^2^ Department of Facial Aesthetics Guarulhos University São Paulo Brazil; ^3^ HOF Pro Academy Goiás Brazil; ^4^ Department of Biological Sciences, Bauru School of Dentistry University of São Paulo Bauru Brazil; ^5^ Department of Biochemistry, Federal University of São Paulo Paulista School of Medicine São Paulo Brazil; ^6^ Private Practice São Paulo Brazil; ^7^ Faculty of Medicine University of Chile Santiago Chile; ^8^ Private Practice Buenos Aires Argentina; ^9^ Center for Radiology and Magnetic Resonance Imaging University Clinical Center of Serbia Belgrade Serbia; ^10^ Faculty of Medicine University of Belgrade Belgrade Serbia; ^11^ Department of Plastic Surgery I.M.Sechenov First Moscow State Medical University Moscow Russia; ^12^ Department of Reconstructive and Plastic Surgery Lancet Clinic Moscow Russia; ^13^ Private Practice San Jose Costa Rica; ^14^ Private Practice Durban South Africa; ^15^ ASST Grande Ospedale Metropolitano Niguarda Piazza Dell'ospedale Maggiore Milano Italy; ^16^ Telemedicine and Telepharmacy Centre, School of Medicinal and Health Products Sciences University of Camerino Camerino Italy; ^17^ Private Practice Milan Italy; ^18^ Centre for Cutaneous Research, Blizard Institute Queen Mary University of London London UK; ^19^ Department of Plastic Surgery Vanderbilt University Medical Center Nashville Tennessee USA; ^20^ Department of Plastic and Reconstructive Surgery Guangdong Second Provincial General Hospital Guangzhou Guangdong Province China

**Keywords:** aesthetic medicine, medical education, scientific output, scientific publishing, social media

## Abstract

**Background:**

Aesthetic medicine is rapidly expanding, yet lack of standardized educational pathways led many healthcare providers (HCPs) to rely on social media influencers for education. The scientific validity of the information shared, however, remains uncertain. Objective: To investigate a correlation between social media popularity and academic profile among professionals active in the field of aesthetic medicine.

**Methods:**

HCPs with a presence on social media were identified randomly from the international aesthetic medical field to allow for a wide range of community‐based representation of aesthetic HCPs. For everyone, publicly available data on follower count, publication number, and H‐index were collected and analyzed.

**Results:**

Among professionals (901), 73.1% (*n* = 659) had zero publications but an average following of 56 898 (228489) followers on Instagram, whereas 63.4% (*n* = 267) had zero publications but an average of 79 580 (377423) followers on TikTok. No significant correlation between social media following and scientific output (Instagram: rp = 0.013, *p* = 0.695; TikTok: r*p* = 0.009, *p* = 0.849) was found.

**Conclusions:**

A high number of followers on social media does not reflect a greater academic profile. Educational content shared by influencers may not be evidence‐based, highlighting the urgent need for structured, regulated guidelines in aesthetic medicine education.

## 1 Introduction

Despite slower growth in the aesthetic medical sector with 1% for surgical and 1.5% for minimally invasive procedures, the popularity and acceptance of cosmetic procedures is constantly growing [1]. Aesthetic medicine has become a part of our daily life and concomitantly with the high demand for treatments a high demand has risen for competent aesthetic treatment providers. Therefore, it is understandable that a constant influx of new healthcare providers (HCPs) has been observed trying to establish their career in providing treatments to patients with aesthetic needs.

Unfortunately, to date there are no board‐specific regulations that govern the education or the educational path of aesthetic medicine HCPs, leaving a substantial gap in the education of aesthetic treatment providers [2]. This poses a risk for patients seeking medical care, especially if the HCP does not have the appropriate experience or knowledge to recognize and to treat adverse events [3, 4]. This gap has allowed private institutions and so‐called key opinion leaders to surface and to share their personal knowledge with the aesthetic community via in‐person or via online educational platforms. However, for the motivated and interested learner, this poses an even greater challenge for obtaining unbiased and evidence based knowledge due to the unfiltered communications which to date occur majorly via social media i.e., Instagram and TikTok. Platforms like Instagram and TikTok have become powerful tools for professionals to showcase results, connect with patients, and build international reputations. This paradigm shift has introduced new dimensions of visibility and influence that transcend traditional academic benchmarks such as scientific publications or conference presentations [5, 6].

In their quest for becoming better aesthetic medicine treatment providers, learners are following trends or recommendations of so‐called social media influencers and accept their transmitted messages into their patient care [7]. From a patient's perspective this is a highly unsatisfactory situation because it is unknown whether such knowledge is based on scientific foundations or whether it's just the personal opinion of someone with a high social media following. From an academic profile, a dichotomy may emerge between professionals who prioritize social engagement and those committed to advancing scientific knowledge. This divergence challenges longstanding assumptions that academic productivity and professional credibility go hand in hand.

To date, no study has systematically examined yet whether professionals with greater social media influence also contribute meaningfully to the scientific literature. For this reason, to investigate the merit of social media‐based teachings in aesthetic medicine this study was designed. In specific, the hypothesis was to investigate whether HCPs with a social media presence on Instagram or on TikTok have a robust scientific background as measured by their number of publications and by their H index; the latter is a metric for evaluating the cumulative impact of an author's scholarly output and performance. The correlation between those parameters will provide insights into the clinical merit of knowledge transfer Via social media especially when coming from so‐called influencers [7]. This gap in knowledge is critical, as it may inform future efforts to establish guidelines, speaker selection criteria for educational events, board certified courses, and the development of dual competencies in academic and social media spheres.

## 2 Material and Methods

### 2.1 Study Design

The present cross‐sectional observational study aimed to explore possible associations between social media presence and scientific output among health care providers in the aesthetic medical field. Publicly available data were collected from online sources between December 2024 and June 2025 for the purposes of this investigation. This study was not interventional and did not involve patient participation, as all information was obtained from publicly accessible profiles and databases. Therefore, ethical approval was not required.

### 2.2 Sample and Data Collection

The total sample investigated consisted of *n* = 901 health care providers (HCPs) working clinically in the aesthetic medical field and had a social media presence on either Instagram (www.instagram.com) or TikTok (www.tiktok.com). The HCPs included in this analysis were randomly identified throughout the entire global aesthetic medical field crossing professional educational lines thereby including physicians and nurses alike. No selective inclusion or exclusion criteria were applied to allow for a wide range of study subjects to be included globally and professionally.

In addition, to actively working in the aesthetic medical field and having a social media presence, the included HCPs were investigated for their quantitative scientific output as listed on pubmed (https://pubmed.ncbi.nlm.nih.gov/) and for their individual citation index as defined by their h index. The h index is a metric for evaluating the cumulative impact of an author's scholarly output and performance and is used in this investigation to reflect on the scientific weight of the HCP [8].

HCPs were not included in this analysis if they had no publicly accessible information regarding their scientific output (number of publications, h index) or for their social media presence (account of Instagram or tiktok).

For each HCP, information regarding country of origin, professional specialty, total number of scientific publications, h index, number of followers on Instagram, and numbers of followers on TikTok were collected. The H‐index was obtained from three major scientific platforms: Web of Science, Google Scholar, and/or Scopus. Social media data were obtained from two of the most widely used platforms globally: Instagram and TikTok, based on publicly accessible professional profiles.

### 2.3 Statistical Analysis

The social media following was dichotomously classified into below and above 100 000 social media followers.

Country of origin was classified according to continents into the following: Africa, Asia, Europe (incl. Russia), North America (incl. US and Canada), Oceania (incl. Australia and New Zealand), South America.

Medical profession was classified first into degrees: medical doctor (independent of specialty), nurse/physician assistants/nurse practitioners, dentists, and other allied health care professionals (beauticians cosmetologists etc.). In the next step, each individual's medical profession was subclassified into their respective medical field. (plastic surgery dermatology etc.).

All statistical analyses were performed using SPSS version 23 (IBM, Armonk, NJ, USA). The mean value and the respective standard deviation (mean ×/− 1× SD) alongside frequencies and cross tabulations were used to describe the sample and the conducted computations. Bivariate correlations (Pearson) were used to identify associations between social media following and scientific output, with results being considered statistically significant at a probability level of *p* ≤ 0.05.

## 3 Results

### 3.1 Sample Characteristics

#### 3.1.1 Demographic Data

**FIGURE 1:**
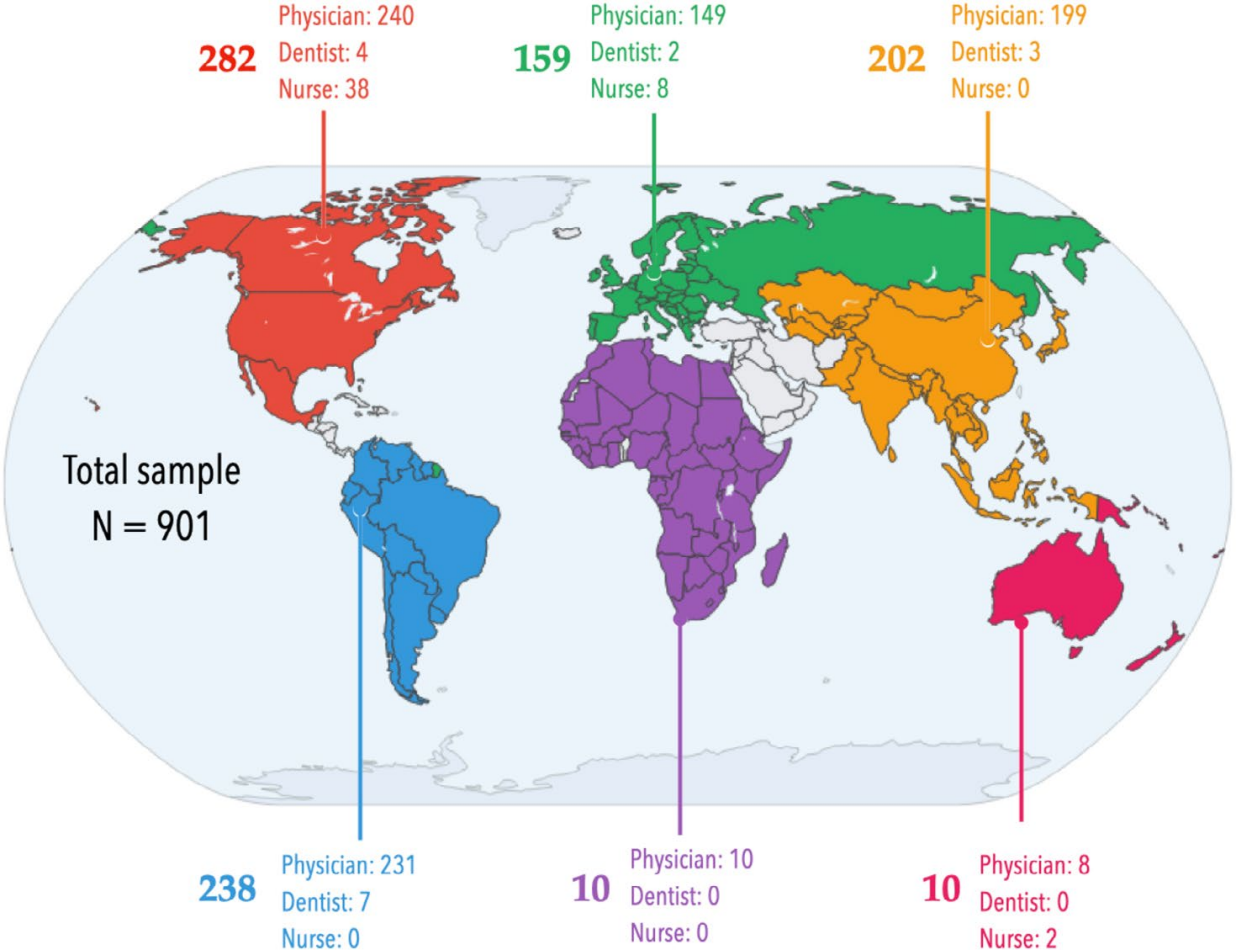
Geographic distribution and professional specialties of the 901 speakers included in the study.

**FIGURE 2:**
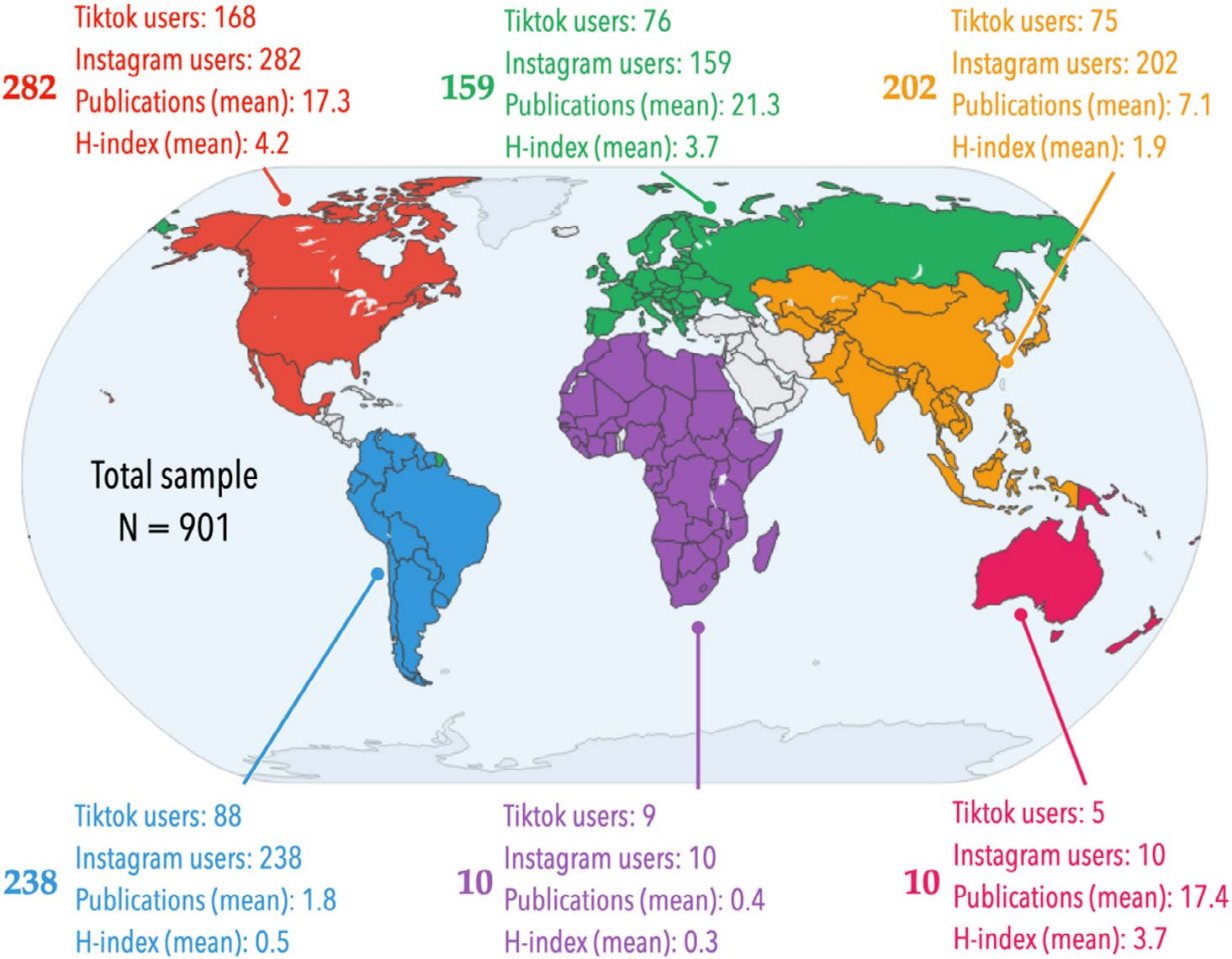
Geographic distribution of social media usage and scientific output among the 901 professionals included in the study. For each region, the number of Instagram and TikTok users is reported alongside the mean number of indexed publications and mean H‐index.

The conducted search identified a total of *n* = 901 HCPs fulfilling the inclusion criteria of this study. Of those *n* = 10 (1.1%) were from Africa, 202 (22.4%) were from Asia, 159 (17.6%) were from Europe (incl. Russia), 282 (31.3%) were from North America, 10 (1.1%) were from Oceania, and 238 (26.4%) were from South America. (Figure 1). Of the *n* = 901, 837 (92.9%) were medical doctors. (51.6% plastic surgery, 28.9% dermatology, 10.8% aesthetic medicine, 1.7% other specialties), 48 (5.3%) were nurses/nurse practitioners/physician assistants, and 16 (1.8%) were dentists (Figure 2).

#### 3.1.2 Social Media Analysis

The average number of followers on social media was 88 261 (304333) [range: 0–4,6 Mio] on Instagram and was 120 702 (561625) [range: 0–8,5 Mio] on TikTok; only 46.7% of the HCPs had both an account on Instagram and TikTok. Of the *n* = 901 HCPs investigated, 84.4% had below 100 000 followers on Instagram and 84.8% had below 100 000 followers on TikTok.

#### 3.1.3 Scientific Output Analysis

**FIGURE 3:**
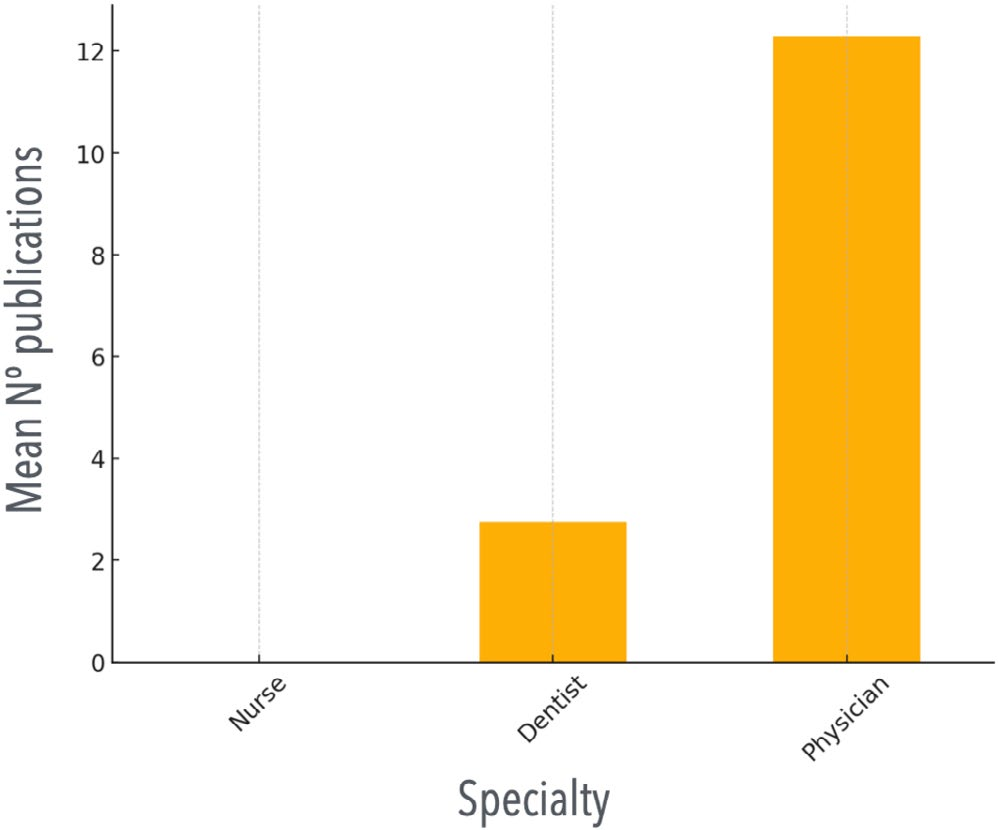
Mean number of indexed publications by specialty among the 901 professionals included in the study.

The total number of scientific publications cumulatively produced by the sample investigated was 10 325 publications with An average of 11.46 (62.45) per HCP. Physicians (independent of specialty) had a mean of 12.3 (64.7) [range: 0–1274] publications, dentists had an average of 2.8 (8.4) publications [range: 0–32], whereas nurses had zero publications in the sample investigated. The average h index per study participant was 2.61 (7.80) with physicians dentists nurses having an h index of 2.80 (8.1) [range: 0–77] 0.75 (2.2) [range: 0–8] zero, respectively. The scientific output per region of practice for the investigated HCPs is shown in Figure 3.

### 3.2 Relationship Between Social Media Following and Number of Publications

**FIGURE 4:**
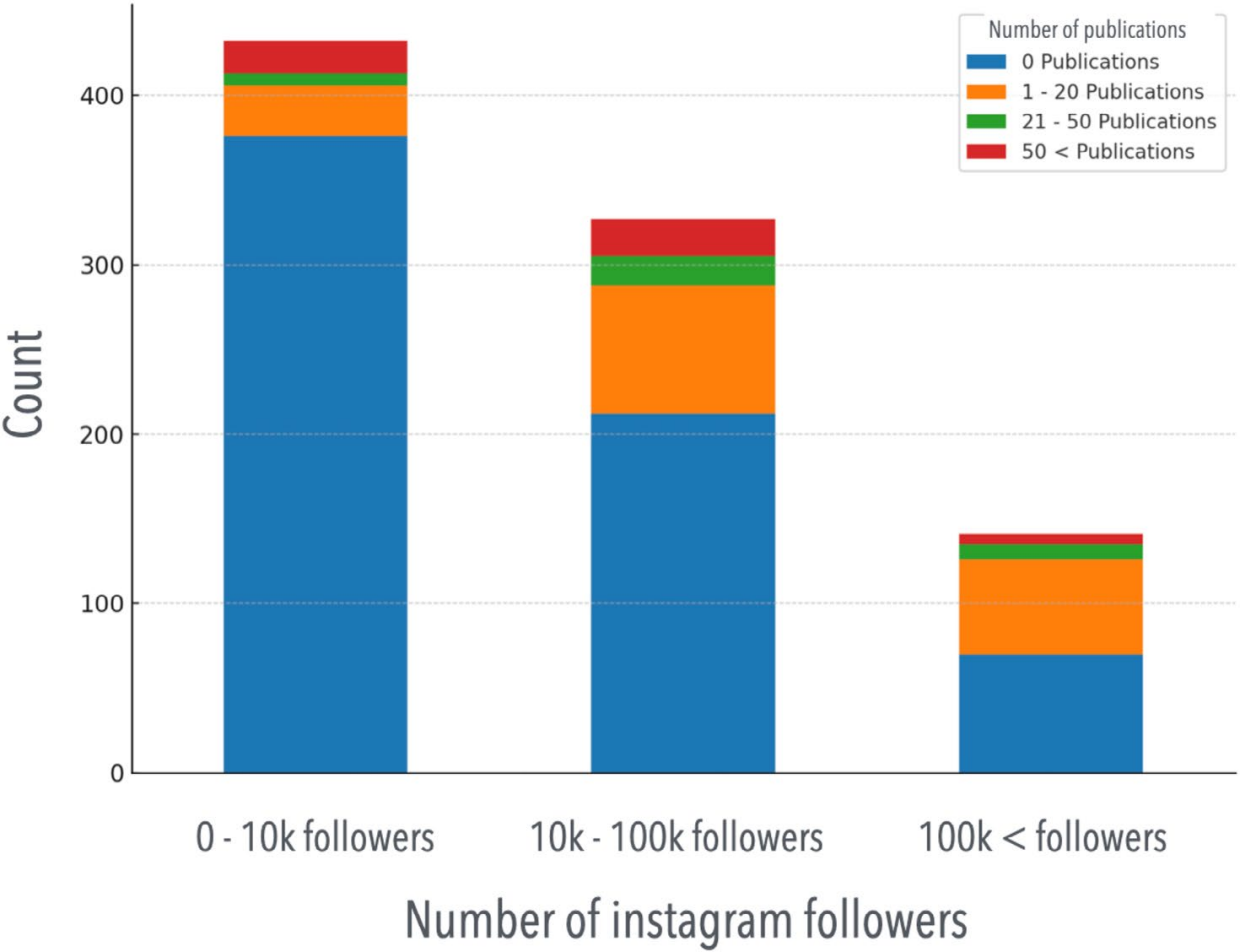
Distribution of professionals according to the number of Instagram followers and indexed publications. The stacked bars represent the proportion of participants in each follower category (< 10 k, 10–100 k, and > 100 k) classified by the number of indexed publications (0, 1–20 , 21–50 , and > 50).

**FIGURE 5:**
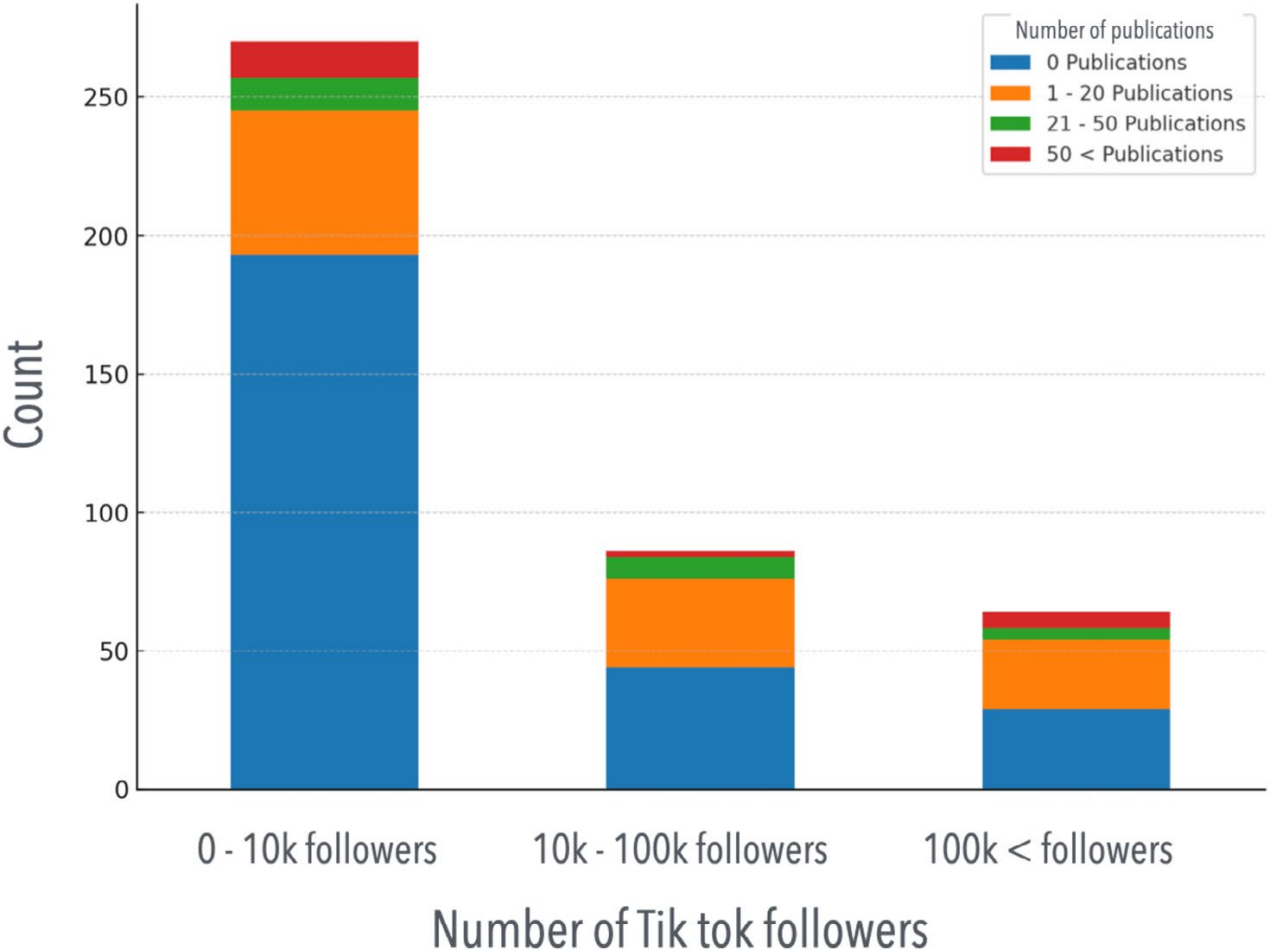
Distribution of professionals according to the number of Tik tok followers and indexed publications. The stacked bars represent the proportion of participants in each follower category (< 10 k, 10–100 k, and > 100 k) classified by the number of indexed publications (0, 1–20 , 21–50 , and > 50).

Of the *n* = 901 HCPs included in this study, 73.1% (*n* = 659) had zero publications but an average following of 56 898 (228489) followers on Instagram (Figure 4) whereas 63.4% (*n* = 267) had zero publications but an average of 79 580 (377423) followers on TikTok (Figure 5). Stratifying the following on social media into below vs. above 100 000 followers it was revealed that for Instagram the average number of publications was 12.1 (67) [range: 0–1274] vs. 7.84 (18) [range: 0–126] and for TikTok it was 8.61 (30) [range: 0–279] vs. 14.2 (34) [range: 0–167], respectively.

The h index for those with less vs. more than 100 000 followers on Instagram was 2.49 (8.0) [range: 0–77] vs. 3.26 (6.5) [range: 0–46] and was on TikTok 2.77 (6.9) [range: 0–62] vs. 4.75 (9.0) [range: 0–46], respectively.

Performing bivariate correlations, no statistically significant associations were identified when investigating the relationship between number of publications vs. social media following for both Instagram (r_p_ = 0.013 with *p* = 0.695) and for TikTok (r_p_ = 0.009 with *p* = 0.849).

## 4 Discussion

This study aimed to investigate the relationship between social media following and scientific output as measured by the number of peer‐reviewed. and pubmed‐indexed publications and by the h index of the respective authors. The results of this cross‐sectional investigation revealed that of the *n* = 901 HCPs included in this study, 73.1% (*n* = 659) had zero publications but an average following of 56 898 (228489) followers on Instagram whereas 63.4% (*n* = 267) had zero publications but an average of 79 580 (377423) followers on TikTok. When performing correlation analyses to identify if a higher following on social media is directly related to a greater scientific gravitas, it was identified that no statistically significant associations were detected when investigating the relationship between number of publications vs. social medial following for both Instagram (r_p_ = 0.013 with *p* = 0.695) and for TikTok (r_p_ = 0.009 with *p* = 0.849). These results indicate that by the methodology applied in this study a greater following on social media does not necessarily indicate that the online messages and teachings conveyed are proportionally supported by the scientific gravitas of the HCP. Instead, it could be inferred that due to the absence of a positive correlation the number of social media followers does not underscore the quality of the education nor does the online popularity reflect on evidence‐based education in the aesthetic medical field. Other factors, most likely lead to social media fame which are most likely disconnected and not related to the work of the respective HCPs in the scientific field. Moreover, it should be considered as a fundamental consideration that evidence‐based medicine in aesthetic medicine is currently lacking both in literature and in formal guidelines [9].

This finding, albeit not surprising in outcome, represents a substantial challenge in the entire aesthetic medical field: how should a novice aesthetic treatment provider structure their educational path if no government‐derived or board‐approved education exists in aesthetic medicine? In addition, who should novice HCPs trust when it comes to their education if no external regulatory institution governs what aesthetic treatment providers should know, do, or be able to execute? [10, 11] Should only universities be responsible for their education? This lack of structure drives young HCPs supported by the technological advancements of this day and age to social media, where educational content is constantly and freely available and with a couple of clicks, an entire aesthetic procedure is reviewed and demonstrated. The decision regarding whom to follow is largely influenced by the number of followers which reflects on social media fame and the provided content. Based on the results of this investigation, it was revealed that even if an HCP has a high social media influence (as reflected by the numbers of followers), the education provided might not be supported by a high scientific output which in turn reflects on the evidence‐based knowledge offered. This gap may be responsible for two main outcomes. First, it fosters the emergence of a group of physicians, dentists, and nurses who are highly active on social media yet not academically engaged. This diverges from what occurs in more traditional medical specialties, where a doctor's reputation is closely tied to their academic profile, reflecting commitment to both research and clinical practice. Second, it creates a group of professionals striving to advance the scientific foundation of the field, yet who remain less visible on social platforms. The question which reflects this thought, often posed in a non‐official way, is: “Would you prefer to be operated for a heart transplant by the most famous cardio surgeon influencer, or by the most skilled with the highest peer‐review scientific background one?”

To continue with, when organizing international and national conferences, speakers are often called by sponsors and key opinion leaders for their social media influence rather than their scientific background [12, 13]. On this regard, the outcomes of our study are important to be taken into consideration by conference organizers. These findings underscore the need to balance speaker selection criteria: social media influence alone does not necessarily reflect scholarly expertise. And, similarly, the preference of speakers with a strong academic background rather than sponsored key opinion leaders could lead to a chained movement where agencies themselves would prefer key opinion leader experts in the field for both clinic and knowledge rather than social media appearance.

In the best of all worlds, a high social media influence of an HCP providing educational content in aesthetic medicine should have a high scientific profile and should provide evidence‐based education based on their scientific work. In other words, our idealistic scenario should embrace teachers and professors with these criteria: a high (or at least present) h index with scientific article production only peer review; scientific article production in non‐predatory journals with at least some Q1–Q2 presence; avoidance of self‐made books without peer review; avoidance of self‐made protocols without scientific validation but divulgated through social media; a strict ethical adherence to deontological regulations; a university background. In such an idealistic scenario, young HCPs could trust that popular social media figures are popular because of their scientific work, which is reflective of unbiased, evidence‐based (not eminence‐based), and neutral education. However, in reality a high social media following is based on almost everything else rather than a high scientific profile with factors like glamour, elite, wealth, lifestyle, humor, and dancing skills to be of greater importance [7]. Unfortunately, the popularity on social media drives education in aesthetic medicine and young HCPs gravitate toward such high‐profile accounts to obtain their knowledge in aesthetic medicine. This is undoubtedly a pessimistic scenario, but the real one that we should deal with [13].

Finally, a consideration should be posed, on the type of HCP: Physicians demonstrated higher publication rates, which may reflect their historically greater involvement in academic networks and research infrastructure. Dentists, in contrast, represent a group that has been more recently integrated into aesthetic medicine in some countries [14], which may partially explain their lower representation in indexed literature. Nurses' absence from indexed literature warrants several attentions, as it may indicate barriers to scholarly inclusion despite their growing clinical role in aesthetics [15]. Similarly, TikTok's variable adoption (highest in North America/Europe; lowest in Asia/South America) may reflect cultural preferences or platform‐specific algorithms favoring certain regions.

Our study is the first in the literature to attempt a combined analysis of academic productivity and social media presence among healthcare professionals in aesthetic medicine. For this reason, it may be open to critique. Nonetheless, this provocative effort is necessary to initiate a call for the development of stringent guidelines in aesthetic medicine. These should be defined by scientific bodies in order to prevent clinical complications and to enhance patient safety [13].

This study is not free of limitations which should be mentioned: first, the HCP accounts investigated were randomly selected and did not follow a country‐specific or discipline‐specific pattern. The aesthetic medical community is larger than the *n* = 901 accounts investigated, but given the possibilities of this investigation, only this sample was considered. Including other social media accounts might have resulted in a different outcome, and future studies will need to expand on the findings presented herein. Second, the number of publications and the h index were used in this investigation to describe the scientific productivity and quality of the investigated HCPs. It could be very likely that some authors have published in a different field or topic rather than aesthetic medicine; this would artificially inflate and misdirect the analyses conducted. However, scientific publishing follows the same rules in various medical fields, and correct scientific conduct is the basis for every publication. Therefore, the number and the h index can be used as a surrogate parameter to reflect on the scientific gravitas as described above. Third, the social media accounts investigated are suggested to also provide aesthetic medicine educational content. This assumption is based on the review of social media accounts, but it cannot be 100% excluded that non‐educational content is released in some posting, diluting the overall educational message of the included. HCP.

## 5 Conclusions

This study reveals a marked disconnection between social media influence and scientific academic profile among aesthetic medicine professionals. Despite the growing use of platforms like Instagram and TikTok for educational purposes, the absence of a correlation between social media following and academic productivity raises concerns about the reliability of such content. In a field lacking formal educational standards, young HCPs are often guided by popularity rather than peer‐reviewed knowledge. Ideally, social media educators should also be academically active, ensuring that what they share is grounded in evidence rather than personal branding. Our findings suggest this is rarely the case. Scientific societies and academic institutions must be responsible and take action in defining educational standards, professors'/teachers' selection criteria, and conference speaker selection criteria. Our study is the first in literature to provide an analysis of correlation between academic and social media dimensions in aesthetic medicine and should serve as a catalyst for critical reflection and regulatory development (other than more evidence‐based medicine and guidelines, but this is a long call we ask for).

## Funding

The authors have nothing to report.

## Consent

The authors have nothing to report.

## Conflicts of Interest

The authors declared no conflicts of interest.

## Data Availability

The data that support the findings of this study are available from the corresponding author upon reasonable request.
